# Rate of residual neuromuscular block using single-dose rocuronium in general anesthesia for ENT surgery: a retrospective cohort study

**DOI:** 10.1186/s12871-023-02027-x

**Published:** 2023-04-01

**Authors:** Orlando Carrillo-Torres, María Guadalupe Pliego-Sánchez, Víctor Joshua Pérez-Muñoz, Jennifer Sánchez-Jurado, Verónica Camacho-Vacherón, José Damián Carrillo-Ruíz

**Affiliations:** 1 Anesthesiology Service at Mexico General Hospital, Mexico City, Mexico; 2 Research Direction & Neurosurgery Service at Hospital General de México, Mexico City, Mexico; 3 Neuroscience Coordination of Psychology Faculty at Mexico Anahuac University, Av. Anahuac 46, Lomas Anahuac, Naucalpan de Juárez, Estado de México, 52786 Mexico

**Keywords:** Neuromuscular block agents, Residual neuromuscular block, Train-of-four, RNMA, RNMB, TOF, TOFR

## Abstract

**Introduction:**

NMB facilitates intubating conditions in general anesthesia. However, it is associated with significant residual postoperative paralysis and morbidity.

**Objective:**

To investigate the rate of underdiagnosed residual NMB based on two TOFR criteria (< 0.91 and < 1.00).

**Methods:**

We performed a retrospective study adhering to STROBE guidelines. We included patients undergoing ENT surgery using single-dose neuromuscular block for balanced general anesthesia from June to December 2018. We collected demographic and anthropometric data, ASA score, NMBA dose, TOFR recordings at 5, 30 and 60 min and end of the surgery, anesthesia and surgery time, and administration of reversal agent. Statistical analysis included descriptive and dispersion measures statistics, curve and cross tables for residual NMB on different TOFR criteria with sub-analysis for AR, RR, and OR in patients over 65 years old.

**Results:**

We included 57 patients, mean age 41; 43 females and 14 males. Mean anesthetic and surgical time were 139.4 and 116.1 min, respectively. All the patients received rocuronium under a mean ponderal single-dose of 0.48 mg/kg. Residual NMB rates were 29.9 and 49.1% for a TOFR < 0.91 and < 1.00, respectively. Older adults had an OR of 6.08 for residual NMB.

**Conclusions:**

The rate of residual NMB was 29.9 to 49.1%, depending on the criteria used (TOFR < 0.91 and < 1.00, respectively). Patients above 65 years old had an increased risk of residual NMB (6.08 OR) and clinical symptoms related to residual NMB (11.75 OR). We recommend future research aiming to provide a specific surveillance protocol for patients above 65 years old, including shorter-action NMB, early reversal, and prolonged surveillance using the TOFR criteria of < 1.00 to identify patients at risk of residual NMB readily.

**Supplementary Information:**

The online version contains supplementary material available at 10.1186/s12871-023-02027-x.

## Highlights

• The rate of residual NMB in our practice was in the range of 29.9 to 49.1% depending on the criteria used (TOFR < 0.91 or TOFR < 1.00, respectively).

• Patients above 65 years old are at an increased risk of residual NMB (6.08 OR) due to increased NMBA duration of action, as demonstrated by the curves of NMB during surgery.

• Older patients also had an increased risk (OR 11.75) of clinical symptoms related to the residual neuromuscular block.

• We recommend providing specific surveillance protocols for patients above 65 years-old, including the use of shorter-action NMB, early reversal, and prolonged surveillance in the postoperative period.

• We recommend using the TOFR criteria of < 1.00 to readily identify more patients at risk of residual neuromuscular block, especially in older people.

• We recommend the use of reversal agents in patients at high risk of residual neuromuscular block, such as patients above 65 years-old, especially in those cases where patients have difficultness for reaching a TOFR criterion of 1.00 because of an increased risk of residual neuromuscular block and its complications.

**Supplementary Information:**

The online version contains supplementary material available at 10.1186/s12871-023-02027-x.

## Introduction

Neuromuscular block agents facilitate intubating conditions in general anesthesia patients by immobilizing vocal cords [1, 17]. However, they are associated with significant residual postoperative paralysis and morbidity [3]. Complete clearance of the agent’s action is essential to safe recovery in the postanesthetic care unit, but it is sometimes incomplete even after using reversal agents [3, 8]. Therefore, postoperative complications remain commonly derived from residual neuromuscular blockade [3, 8]. Acceleromyography is the standard neuromuscular block monitoring in clinical practice; it relies on the train-of-four nerve stimulation pattern recommendation: or the ratio of the fourth twitch to the first twitch T4/T1 ratio [11]. A train-of-four ratio of 0.7–0.9 is associated with impaired airway protective reflexes, upper airway obstruction, and postoperative hypoxemia [6, 12]. Therefore, full recovery of neuromuscular function should be present at the time of tracheal extubations [12]. Recent research argues that specific devices using raw acceleromyography may overestimate neuromuscular recovery and that the train-of-four ratio must recover to 0.99 or even 1.00 to exclude residual paralysis in this setting [5, 12, 15]. Our study aims to investigate the rate of the underdiagnosed residual neuromuscular block based on these two train-of-four ratio criteria (< 0.91 and < 1.00) to provide a framework to improve patient safety during surgery. Additionally, using crosstables, we calculated the relative risk, absolute risk, and Odds ratios for patients over 65 as compared to patients under 65 on the risk of residual neuromuscular block.

## Methods

We performed a retrospective observational cross-sectional study adhering to STROBE guidelines. The protocol was approved by the General Hospital of Mexico Ethics and Research Committee by the number: DI/20/101/03/77; informed consent was waived because of being a retrospective study. We reviewed the clinical files of patients undergoing Ear-Nose-Throat (ENT) surgery from June 2018 to December 2018 in the General Hospital of Mexico. Inclusion criteria were adult patients (over 18 years) with an American Society of Anesthesiologists score (ASA) risk between I and III, having TOFR monitoring throughout balanced general anesthesia (induction with intravenous fentanyl and propofol and anesthetic maintenance with inhalational sevoflurane) using neuromuscular block with rocuronium (ponderal dose 0.3–1.2 mg/kg) at a single dose. Exclusion criteria considered any muscle relaxant other than rocuronium, patients requiring additional muscle relaxant doses during surgery, muscle-related diseases or allergies, magnesium sulfate or antiepileptics medication, or illnesses affecting TOFR evaluation and incomplete medical records. We collected demographic and anthropometric data, ASA score, NMBA and dose, TOFR registers at 5 min, 30 min, 60 min, and end of the surgery, anesthesia time in minutes, surgery time, and administration of reversal agent. We performed descriptive statistics (frequencies and percentages) for demographic data; we used mean dispersion measures (mean, standard deviation, 95% CI) for continuous variables. We used graphics to demonstrate the curve of NMB during surgery and residual NMB as the outcome in percentages. We used cross tables for residual NMB using two criteria, including TOFR < 0.91 or TOFR < 1.00. We made a sub-analysis for residual NMB using both criteria on patients under and above 65 years old. Table 1 includes the dataset of the whole sample and variables studied for statistical analysis.

**Table 1:** Sample and variables dataset

ID	AGE	AGE ABOVE 65	GENDER	WEIGHT	HEIGHT	BMI	ASA	MUSCLE RELAXANT	DOSE	PONDERAL DOSE	TOF 5Min	TOF 30Min	TOF 60Min	TOF EOS	ANESTHESIA TIME	SURGERY TIME
1	25	NO	F	50.0	155	20.81	1	ROCURONIUM	40	0.80	0	3	82	89	130	110
2	40	NO	F	52.0	155	21.64	1	ROCURONIUM	30	0.58	1	1	29	82	120	90
3	40	NO	F	73.0	145	34.72	2	ROCURONIUM	30	0.41	0	1	5	99	70	60
4	27	NO	M	73.0	170	25.26	2	ROCURONIUM	40	0.55	2	64	126	126	165	150
5	56	NO	F	84.0	152	36.36	3	ROCURONIUM	40	0.48	2	38	74	89	110	95
6	42	NO	F	50.0	158	20.03	2	ROCURONIUM	30	0.60	0	10	27	101	76	64
7	36	NO	M	75.0	168	26.57	2	ROCURONIUM	40	0.53	0	25	62	62	120	90
8	30	NO	F	55.0	156	22.60	1	ROCURONIUM	30	0.55	2	2	158	158	220	200
9	67	YES	F	63.0	153	26.91	2	ROCURONIUM	30	0.48	0	1	33	108	110	80
10	61	NO	F	57.0	160	22.27	2	ROCURONIUM	25	0.44	0	30	53	61	60	50
11	42	NO	M	76.0	170	26.30	3	ROCURONIUM	40	0.53	0	1	58	90	160	150
12	20	NO	F	63.0	155	26.22	2	ROCURONIUM	30	0.48	0	1	62	126	140	130
13	28	NO	F	58.0	151	25.44	1	ROCURONIUM	30	0.52	0	1	31	92	180	160
14	33	NO	M	55.0	154	23.19	1	ROCURONIUM	30	0.55	0	1	94	95	120	100
15	30	NO	F	85.0	170	29.41	2	ROCURONIUM	35	0.41	0	1	62	92	70	45
16	54	NO	F	60.0	148	27.39	2	ROCURONIUM	30	0.50	20	49	91	98	260	230
17	57	NO	F	63.0	160	24.61	2	ROCURONIUM	35	0.56	0	0	36	73	90	60
18	54	NO	F	62.0	145	29.49	2	ROCURONIUM	35	0.56	0	0	17	98	80	40
19	18	NO	F	45.0	150	20.00	1	ROCURONIUM	30	0.67	2	27	97	102	140	120
20	69	YES	F	62.0	165	22.77	2	ROCURONIUM	30	0.48	35	2	57	87	170	150
21	24	NO	M	65.0	166	23.59	1	ROCURONIUM	30	0.46	2	53	87	109	85	70
22	38	NO	F	60.0	144	28.94	2	ROCURONIUM	30	0.50	9	16	81	144	160	135
23	30	NO	M	82.0	172	27.72	2	ROCURONIUM	40	0.49	20	2	35	59	105	90
24	80	YES	F	49.0	150	21.78	2	ROCURONIUM	30	0.61	3	20	80	96	128	108
25	78	YES	F	65.0	160	25.39	2	ROCURONIUM	20	0.31	0	0	17	84	120	90
26	22	NO	M	62.0	178	19.57	3	ROCURONIUM	40	0.65	0	2	70	100	210	180
27	46	NO	F	65.0	162	24.77	2	ROCURONIUM	30	0.46	0	25	60	100	300	240
28	35	NO	F	78.0	152	33.76	2	ROCURONIUM	30	0.38	0	0	50	50	100	90
29	67	YES	F	73.0	160	28.52	2	ROCURONIUM	35	0.48	0	2	26	90	190	130
30	77	YES	M	56.0	161	21.60	2	ROCURONIUM	30	0.54	0	30	50	92	175	140
31	41	NO	F	49.0	148	22.37	3	ROCURONIUM	40	0.82	0	27	58	90	160	140
32	38	0.00	F	63.0	155	26.22	2	ROCURONIUM	30	0.48	0	13	45	100	220	180
33	54	0.00	F	101.0	157	40.98	3	ROCURONIUM	50	0.50	0	0	3	60	150	120
34	55	0.00	F	64.0	151	28.07	2	ROCURONIUM	30	0.47	0	50	100	100	230	200
35	53	0.00	M	84.0	174	27.74	2	ROCURONIUM	35	0.42	0	70	76	80	80	60
36	25	0.00	F	85.0	160	33.20	1	ROCURONIUM	30	0.35	3	20	80	100	110	90
37	48	0.00	F	74.0	152	32.03	2	ROCURONIUM	30	0.41	0	40	80	100	180	160
38	33	0.00	F	61.0	156	25.07	2	ROCURONIUM	30	0.49	16	72	100	100	150	120
39	21	0.00	F	81.0	166	29.39	2	ROCURONIUM	35	0.43	0	13	74	100	126	95
40	61	0.00	F	67.0	157	27.18	3	ROCURONIUM	30	0.45	3	20	58	102	175	130
41	37	0.00	F	67.0	145	31.87	2	ROCURONIUM	30	0.45	0	28	96	100	90	70
42	18	0.00	M	102.0	171	34.88	1	ROCURONIUM	50	0.49	0	64	97	113	200	180
43	50	0.00	F	63.0	159	24.92	2	ROCURONIUM	30	0.48	4	2	62	110	235	195
44	49	0.00	F	73.3	165	26.92	2	ROCURONIUM	30	0.41	2	5	100	100	120	100
45	48	0.00	M	88.0	162	33.53	3	ROCURONIUM	30	0.34	2	48	95	95	80	60
46	52	0.00	F	68.0	145	32.34	2	ROCURONIUM	35	0.51	4	17	45	64	60	45
47	23	0.00	M	98.0	181	29.91	2	ROCURONIUM	40	0.41	2	5	88	110	100	75
48	42	0.00	F	79.0	165	29.02	2	ROCURONIUM	30	0.38	4	14	76	87	75	58
49	41	0.00	F	52.0	150	23.11	1	ROCURONIUM	30	0.58	4	3	91	101	120	100
50	59	0.00	F	71.0	167	25.46	2	ROCURONIUM	50	0.70	3	20	59	91	240	220
51	23	0.00	F	56.0	168	19.84	1	ROCURONIUM	30	0.54	20	40	60	102	80	65
52	43	0.00	M	77.0	159	30.46	2	ROCURONIUM	30	0.39	0	0	62	98	90	75
53	24	0.00	F	50.0	158	20.03	1	ROCURONIUM	30	0.60	0	25	55	105	305	288
54	51	0.00	F	52.0	156	21.37	3	ROCURONIUM	30	0.58	0	18	90	116	125	80
55	30	0.00	F	55.0	158	22.03	2	ROCURONIUM	20	0.36	0	20	146	146	72	45
56	18	0.00	M	69.0	164	25.65	2	ROCURONIUM	30	0.43	0	12	92	112	130	100
57	16	0.00	F	65.0	170	22.49	1	ROCURONIUM	40	0.62	0	13	35	112	80	122

ID	ANESTHESIA AT 90 MIN	REVERSION	TOF 5MIN DX < 0.91	TOF 5MIN DX < 1.0	TOF 30MIN DX < 0.91	TOF 30MIN DX < 1.0	TOF 60MIN DX < 0.91	TOF 60MIN DX < 1.0	TOF EOS DX < 0.91	TOF EOS DX < 1.0	REVERSAL DRUG	DX < 0.91 EOS	DX < 1.0 EOS	SYMPTOMS	COMPLICATIONS
1	YES	NO	NMB ON	NMB ON	NMB ON	NMB ON	NMB ON	NMB ON	NMB ON	NMB ON		UNDERDIAGNOSIS	UNDERDIAGNOSIS		
2	YES	NO	NMB ON	NMB ON	NMB ON	NMB ON	NMB ON	NMB ON	NMB ON	NMB ON		UNDERDIAGNOSIS	UNDERDIAGNOSIS		
3	NO	NO	NMB ON	NMB ON	NMB ON	NMB ON	NMB ON	NMB ON	NMB OFF	NMB ON		RECOVERED	UNDERDIAGNOSIS		
4	YES	NO	NMB ON	NMB ON	NMB ON	NMB ON	NMB OFF	NMB OFF	NMB OFF	NMB OFF		RECOVERED	RECOVERED		
5	YES	NO	NMB ON	NMB ON	NMB ON	NMB ON	NMB ON	NMB ON	NMB ON	NMB ON		UNDERDIAGNOSIS	UNDERDIAGNOSIS		
6	NO	NO	NMB ON	NMB ON	NMB ON	NMB ON	NMB ON	NMB ON	NMB OFF	NMB OFF		RECOVERED	RECOVERED		
7	YES	NO	NMB ON	NMB ON	NMB ON	NMB ON	NMB ON	NMB ON	NMB ON	NMB ON		UNDERDIAGNOSIS	UNDERDIAGNOSIS		
8	YES	NO	NMB ON	NMB ON	NMB ON	NMB ON	NMB OFF	NMB OFF	NMB OFF	NMB OFF		RECOVERED	RECOVERED		
9	YES	NO	NMB ON	NMB ON	NMB ON	NMB ON	NMB ON	NMB ON	NMB OFF	NMB OFF		RECOVERED	RECOVERED		
10	NO	NO	NMB ON	NMB ON	NMB ON	NMB ON	NMB ON	NMB ON	NMB ON	NMB ON		UNDERDIAGNOSIS	UNDERDIAGNOSIS		LOWER RESPIRATORY TRACT INFECTION
11	YES	NO	NMB ON	NMB ON	NMB ON	NMB ON	NMB ON	NMB ON	NMB ON	NMB ON		UNDERDIAGNOSIS	UNDERDIAGNOSIS	SWALLOWING WEAKNESS	
12	YES	NO	NMB ON	NMB ON	NMB ON	NMB ON	NMB ON	NMB ON	NMB OFF	NMB OFF		RECOVERED	RECOVERED		
13	YES	NO	NMB ON	NMB ON	NMB ON	NMB ON	NMB ON	NMB ON	NMB OFF	NMB ON		RECOVERED	UNDERDIAGNOSIS		
14	YES	NO	NMB ON	NMB ON	NMB ON	NMB ON	NMB OFF	NMB ON	NMB OFF	NMB ON		RECOVERED	UNDERDIAGNOSIS		
15	NO	NO	NMB ON	NMB ON	NMB ON	NMB ON	NMB ON	NMB ON	NMB OFF	NMB ON		RECOVERED	UNDERDIAGNOSIS	SWALLOWING WEAKNESS	
16	YES	NO	NMB ON	NMB ON	NMB ON	NMB ON	NMB OFF	NMB ON	NMB OFF	NMB ON		RECOVERED	UNDERDIAGNOSIS		
17	YES	NO	NMB ON	NMB ON	NMB ON	NMB ON	NMB ON	NMB ON	NMB ON	NMB ON		UNDERDIAGNOSIS	UNDERDIAGNOSIS		
18	NO	YES	NMB ON	NMB ON	NMB ON	NMB ON	NMB ON	NMB ON	NMB OFF	NMB ON	SUGAM MADEX	OVERDIAGNOSED	DIAGNOSED		
19	YES	NO	NMB ON	NMB ON	NMB ON	NMB ON	NMB OFF	NMB ON	NMB OFF	NMB OFF		RECOVERED	RECOVERED		
20	YES	NO	NMB ON	NMB ON	NMB ON	NMB ON	NMB ON	NMB ON	NMB ON	NMB ON		UNDERDIAGNOSIS	UNDERDIAGNOSIS	SWALLOWING WEAKNESS	
21	NO	NO	NMB ON	NMB ON	NMB ON	NMB ON	NMB ON	NMB ON	NMB OFF	NMB OFF		RECOVERED	RECOVERED		
22	YES	NO	NMB ON	NMB ON	NMB ON	NMB ON	NMB ON	NMB ON	NMB OFF	NMB OFF		RECOVERED	RECOVERED		UPPER RESPIRATORY TRACT INFECTION
23	YES	NO	NMB ON	NMB ON	NMB ON	NMB ON	NMB ON	NMB ON	NMB ON	NMB ON		UNDERDIAGNOSIS	UNDERDIAGNOSIS		
24	YES	NO	NMB ON	NMB ON	NMB ON	NMB ON	NMB ON	NMB ON	NMB OFF	NMB ON		RECOVERED	UNDERDIAGNOSIS	SWALLOWING WEAKNESS	
25	YES	NO	NMB ON	NMB ON	NMB ON	NMB ON	NMB ON	NMB ON	NMB ON	NMB ON		UNDERDIAGNOSIS	UNDERDIAGNOSIS		
26	YES	NO	NMB ON	NMB ON	NMB ON	NMB ON	NMB ON	NMB ON	NMB OFF	NMB OFF		RECOVERED	RECOVERED		
27	YES	NO	NMB ON	NMB ON	NMB ON	NMB ON	NMB ON	NMB ON	NMB OFF	NMB OFF		RECOVERED	RECOVERED		
28	YES	YES	NMB ON	NMB ON	NMB ON	NMB ON	NMB ON	NMB ON	NMB ON	NMB ON	SUGAM MADEX	DIAGNOSED	DIAGNOSED		
29	YES	NO	NMB ON	NMB ON	NMB ON	NMB ON	NMB ON	NMB ON	NMB ON	NMB ON		UNDERDIAGNOSIS	UNDERDIAGNOSIS		
30	YES	NO	NMB ON	NMB ON	NMB ON	NMB ON	NMB ON	NMB ON	NMB OFF	NMB ON		RECOVERED	UNDERDIAGNOSIS	SWALLOWING WEAKNESS	
31	YES	NO	NMB ON	NMB ON	NMB ON	NMB ON	NMB ON	NMB ON	NMB ON	NMB ON		UNDERDIAGNOSIS	UNDERDIAGNOSIS		
32	YES	NO	NMB ON	NMB ON	NMB ON	NMB ON	NMB ON	NMB ON	NMB OFF	NMB OFF		RECOVERED	RECOVERED		
33	YES	YES	NMB ON	NMB ON	NMB ON	NMB ON	NMB ON	NMB ON	NMB ON	NMB ON	SUGAM MADEX	DIAGNOSED	DIAGNOSED		
34	YES	NO	NMB ON	NMB ON	NMB ON	NMB ON	NMB OFF	NMB OFF	NMB OFF	NMB OFF		RECOVERED	RECOVERED		
35	NO	NO	NMB ON	NMB ON	NMB ON	NMB ON	NMB ON	NMB ON	NMB ON	NMB ON		UNDERDIAGNOSIS	UNDERDIAGNOSIS		
36	YES	NO	NMB ON	NMB ON	NMB ON	NMB ON	NMB ON	NMB ON	NMB OFF	NMB OFF		RECOVERED	RECOVERED		
37	YES	NO	NMB ON	NMB ON	NMB ON	NMB ON	NMB ON	NMB ON	NMB OFF	NMB OFF		RECOVERED	RECOVERED		
38	YES	NO	NMB ON	NMB ON	NMB ON	NMB ON	NMB OFF	NMB OFF	NMB OFF	NMB OFF		RECOVERED	RECOVERED		
39	YES	NO	NMB ON	NMB ON	NMB ON	NMB ON	NMB ON	NMB ON	NMB OFF	NMB OFF		RECOVERED	RECOVERED		
40	YES	NO	NMB ON	NMB ON	NMB ON	NMB ON	NMB ON	NMB ON	NMB OFF	NMB OFF		RECOVERED	RECOVERED		
41	YES	NO	NMB ON	NMB ON	NMB ON	NMB ON	NMB OFF	NMB ON	NMB OFF	NMB OFF		RECOVERED	RECOVERED		
42	YES	NO	NMB ON	NMB ON	NMB ON	NMB ON	NMB OFF	NMB ON	NMB OFF	NMB OFF		RECOVERED	RECOVERED		
43	YES	NO	NMB ON	NMB ON	NMB ON	NMB ON	NMB ON	NMB ON	NMB OFF	NMB OFF		RECOVERED	RECOVERED		
44	YES	NO	NMB ON	NMB ON	NMB ON	NMB ON	NMB OFF	NMB OFF	NMB OFF	NMB OFF		RECOVERED	RECOVERED		
45	NO	NO	NMB ON	NMB ON	NMB ON	NMB ON	NMB OFF	NMB ON	NMB OFF	NMB ON		RECOVERED	UNDERDIAGNOSIS		LOWER RESPIRATORY TRACT INFECTION
46	NO	NO	NMB ON	NMB ON	NMB ON	NMB ON	NMB ON	NMB ON	NMB ON	NMB ON		UNDERDIAGNOSIS	UNDERDIAGNOSIS	SWALLOWING WEAKNESS	
47	YES	NO	NMB ON	NMB ON	NMB ON	NMB ON	NMB ON	NMB ON	NMB OFF	NMB OFF		RECOVERED	RECOVERED		
48	NO	NO	NMB ON	NMB ON	NMB ON	NMB ON	NMB ON	NMB ON	NMB ON	NMB ON		UNDERDIAGNOSIS	UNDERDIAGNOSIS		
49	YES	NO	NMB ON	NMB ON	NMB ON	NMB ON	NMB OFF	NMB ON	NMB OFF	NMB OFF		RECOVERED	RECOVERED		
50	YES	NO	NMB ON	NMB ON	NMB ON	NMB ON	NMB ON	NMB ON	NMB OFF	NMB ON		RECOVERED	UNDERDIAGNOSIS	SWALLOWING WEAKNESS	UPPER RESPIRATORY TRACT INFECTION
51	NO	NO	NMB ON	NMB ON	NMB ON	NMB ON	NMB ON	NMB ON	NMB OFF	NMB OFF		RECOVERED	RECOVERED		
52	YES	NO	NMB ON	NMB ON	NMB ON	NMB ON	NMB ON	NMB ON	NMB OFF	NMB ON		RECOVERED	UNDERDIAGNOSIS		
53	YES	NO	NMB ON	NMB ON	NMB ON	NMB ON	NMB ON	NMB ON	NMB OFF	NMB OFF		RECOVERED	RECOVERED		
54	YES	NO	NMB ON	NMB ON	NMB ON	NMB ON	NMB OFF	NMB ON	NMB OFF	NMB OFF		RECOVERED	RECOVERED		
55	NO	NO	NMB ON	NMB ON	NMB ON	NMB ON	NMB OFF	NMB OFF	NMB OFF	NMB OFF		RECOVERED	RECOVERED		
56	YES	NO	NMB ON	NMB ON	NMB ON	NMB ON	NMB OFF	NMB ON	NMB OFF	NMB OFF		RECOVERED	RECOVERED		
57	NO	NO	NMB ON	NMB ON	NMB ON	NMB ON	NMB ON	NMB ON	NMB OFF	NMB OFF		RECOVERED	RECOVERED		

This dataset includes the whole sample, and variables studied for statistical analysis

*ASA* American Society of Anesthesiologists score, *BMI* Body Mass Index, *DX < 91* Diagnostic criteria < 91%, *EOS* End Of Surgery, *TOF* Train-Of-Four, *NMB* Neuromuscular Block

### TOFR recording process and documentation

**Fig. 1:**
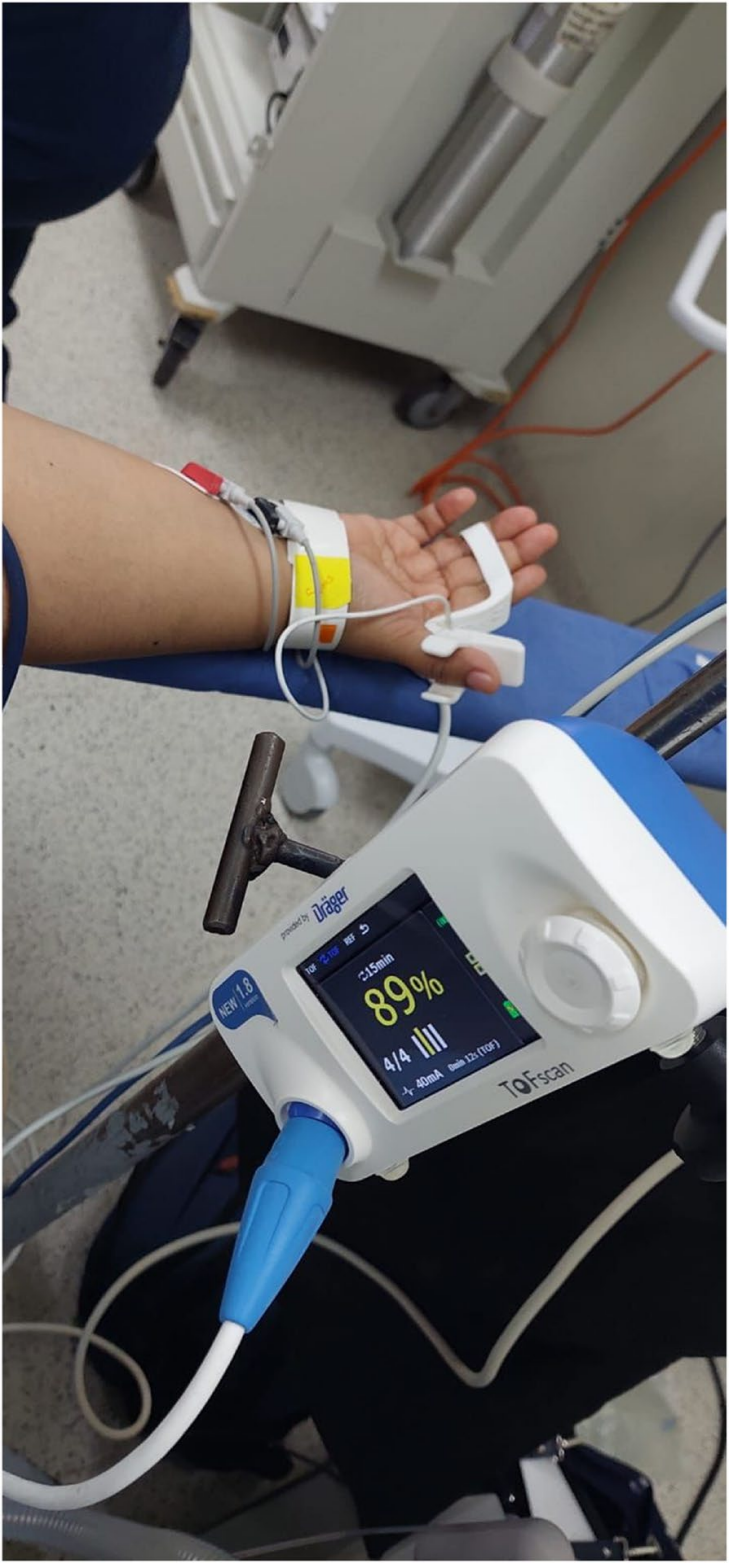
TOFR measurement and electrodes disposition

All TOFR Recordings performed were done using an acceleromyographic portable Drager TOFScan® locating two electrodes on the wrist and the distal forearm of the non-dominant hand to stimulate the first finger abductor and register its movement using the second or third finger as reference (Fig. 1), TOFR measure is performed initially just before starting neuromuscular block, then forward we performed secondary measures at 5, 30 and 60 minutes as well as at the ending of the surgical procedure, where we keep monitoring up until the patient reaches a TOFR of 1.00 or above (raw data) before proceeding to extubate the patient. Given the retrospective nature of the study, the administration of reversal agents was performed based on the treating anesthesiologist’s criteria. All anesthetic events and recordings are documented on the anesthesia sheet, and data extraction for this study was gathered from these patients’ files.

## Results

**Fig. 2:**
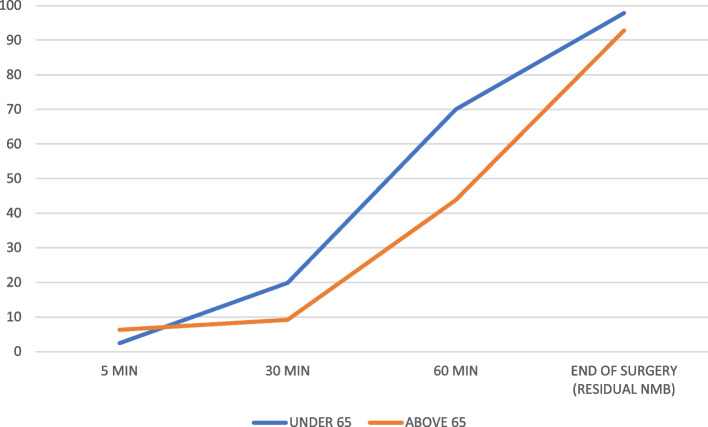
Compares the mean TOFR values in patients below and over 65 years old over time

**Fig. 3:**
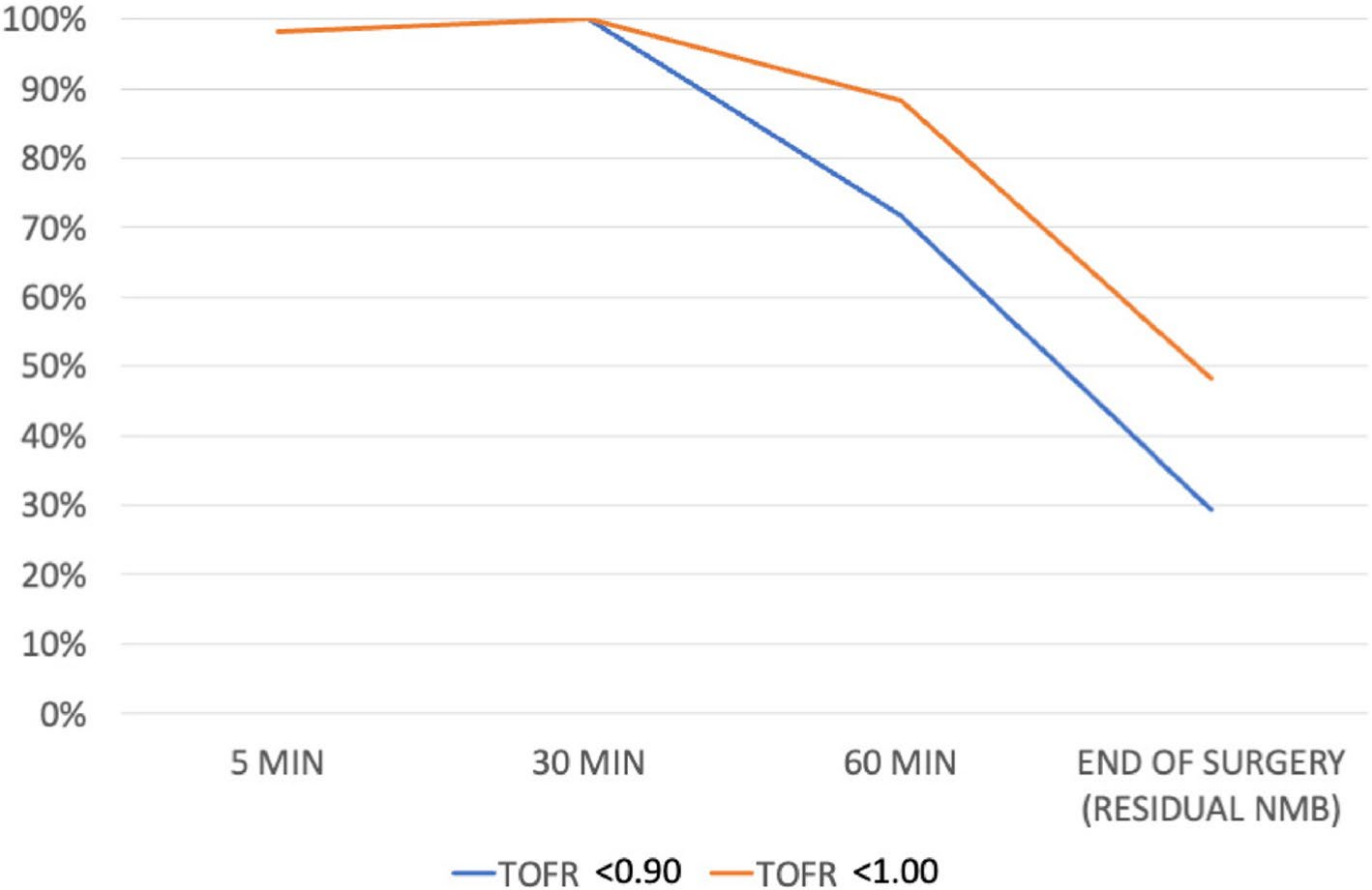
Compares the percentage of NMB over time according to the different TOFR criteria, TOFR < 0.91 or TOFR < 1.00

**Fig. 4:**
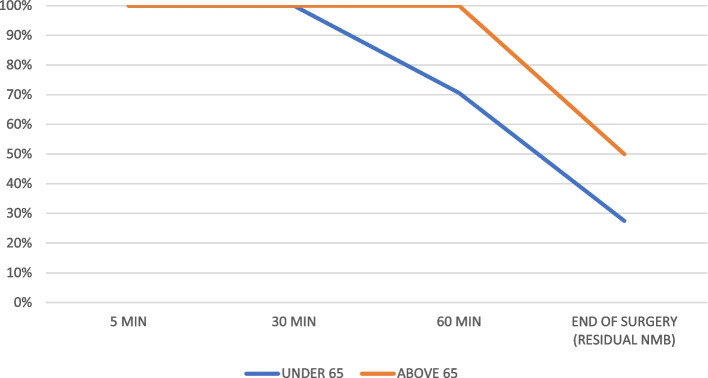
Compares the percentage of NMB in patients under and above 65 years old over time according to TOFR < 0.91

**Fig. 5:**
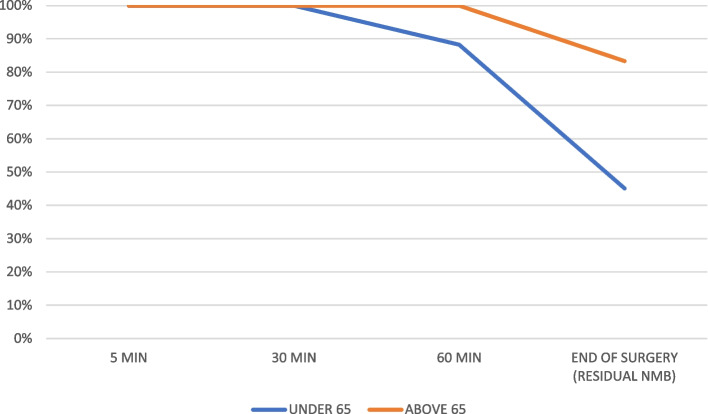
Compares the curve of NMB in patients under and above 65 years old over time according to TOFR < 1.00

**Fig. 6:**
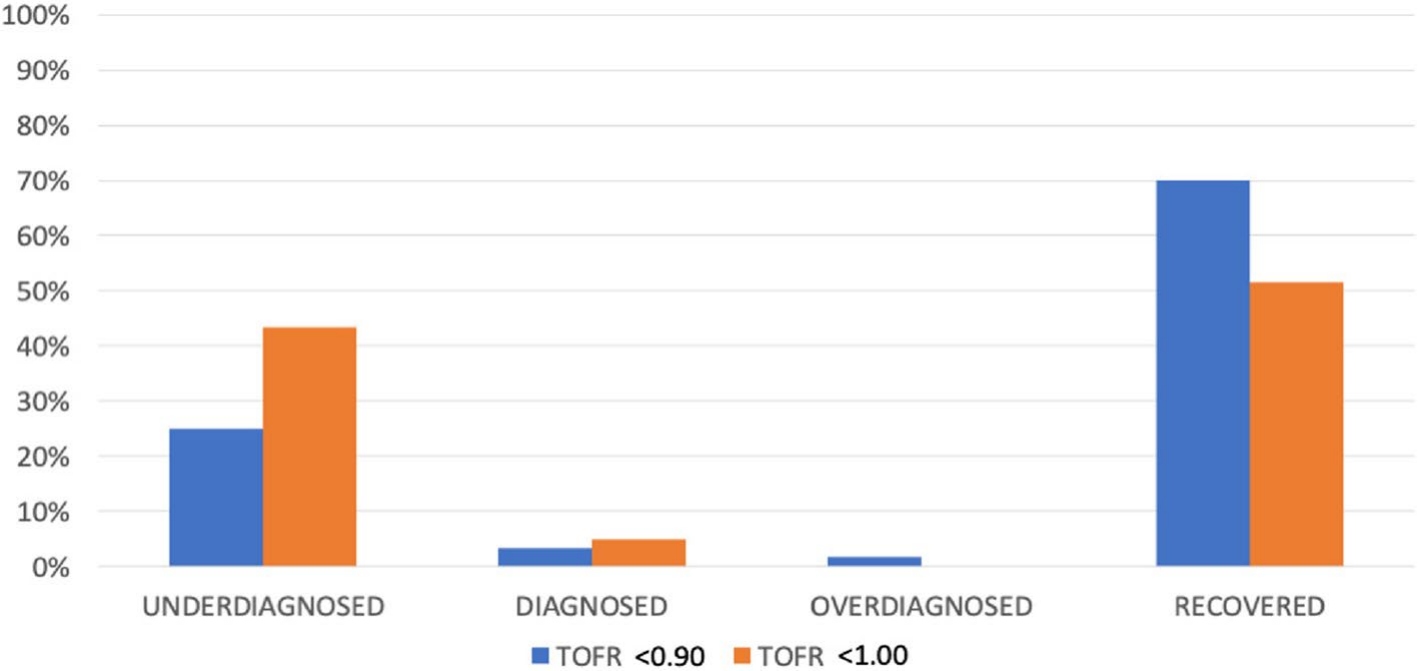
Compares the rates of underdiagnosis, diagnosis, and overdiagnosis of residual NMB to recovered patients according to the different TOFR criterium, TOFR < 0.91 or TOFR < 1.00

We included 57 patients and excluded no patients; 43 females (75.4%) and 14 males (24.6%). The mean age was 41.74 years old (16.62 standard deviation (SD), 95% confidence interval (CI) at 37.33–46.15), and the mean body mass index (BMI) was 26.56 kg/m2 (4.72 SD, 95%CI at 25.30–27.81 95%). Patients more frequently had an ASA score of II (36 patients, 63.2%), followed by I (13 patients, 22.8%), and lastly, III (8 patients, 14.0%). The mean anesthetic time was 139.42 min (59.92 SD, 95%CI at 123.52–155.32), while the mean surgical time was 116.14 min (55.69 SD, 95%CI at 101.36–130.92). All 57 patients received a rocuronium at a mean ponderal single-dose of 0.50 (0.10 SD, 95%CI at 0.47–0.49) mg/kg. Mean TOFR values were as follows: 5 min 0.029 (0.06 SD, 95%CI at 0.01–0.04), 30 min 0.19 (0.20 SD, 95%CI at 0.13–0.24), 60 min 0.67 (0.31 SD, 95%CI 0.59–0.76), and at the end of surgery (outcome) 0.97 (0.20 SD, 95%CI at 0.92–1.03). Figure 2 compares the mean TOFR values in patients below and over 65 years old over time. Figure 3 compares the percentage of NMB over time according to the different TOFR criterium, TOFR < 0.91 or TOFR < 1.00. Figure 4 compares the percentage of NMB in patients under and above 65 years old over time according to TOFR < 0.91. Figure 5 compares the curve of NMB in patients under and above 65 years old over time according to TOFR < 1.00. Figure 6 compares the rates of underdiagnosis, diagnosis, and overdiagnosis of residual NMB to recovered patients according to the different TOFR criterium, TOFR < 0.91 or TOFR < 1.00. The residual NMB in our series was 29.9 to 49.1%, depending on the criteria used (TOFR < 0.91 or TOFR < 1.00, respectively). By subgroup analysis, patients below 65 years old had residual NMB varying from 27.5 to 45.1%, while patients above 65 years old were in the range of 50.0 to 83.3% using the different TOFR criteria (< 0.91 and < 1.00, respectively). Patients under 65 years old had a minimum NMB effect of at least 60 minutes; with almost 74.51% (38/51 patients) of them exceeding 90 minutes; while none of the patients above 65 years old were below 90 minutes, with a minimum of 110 minutes. Using TOFR criteria of < 0.91, cross tables for residual NMB in patients above 65 years old demonstrated an increased Absolute Risk (AR) ratio of 0.22, Relative Risk (RR) ratio of 1.82, and Odds Ratio (OR) of 2.64 as compared to patients under 65 years old. Using TOFR criteria of < 1.00, cross tables for Residual NMB in patients above 65 years old demonstrate an increased Absolute Risk (AR) ratio of 0.38, Relative Risk (RR) ratio of 1.84, and Odds Ratio (OR) of 6.09 as compared to patients under 65 years old. Seven (12.3%) patients had clinical symptoms of residual neuromuscular block presented as swallowing weakness in the postanesthetic recovery room, having a final TOFR below 1.00 before leaving the surgical room, two of them had a TOFR below 0.91, and surprisingly one of them had a TOFR of 0.64. The sensibility of a TOFR < 0.91 to detect clinical signs of residual neuromuscular block was 43%, the specificity was 72%, the Positive Predictive Value (PPV) was 18%, and the Negative Predictive Value (NPV) was 90%, with an AR of 0.07, a RR of 1.76 and an OR of 1.93. The sensibility of a TOFR < 1.0 to detect clinical signs of residual neuromuscular block was 100%, the specificity was 58%, the PPV was 25%, and the NPV was 100% with an AR of 0.25, and non-determined RR and OR because of no false negative cases. The AR for patients over 65 with clinical symptoms of residual neuromuscular block was 0.42, with a RR of 6.37 and an OR of 11.75. None of the patients required to be re-intubated, nor did they have a respiratory compromise. None of the patients receiving sugammadex presented clinical symptoms of residual neuromuscular block. Four patients presented complications, two (3.5%) had upper respiratory tract infections, and the other two (3.5%) had lower respiratory tract infections during their in-hospital stay. One of the patients with a lower respiratory tract infection had a final TOFR of 0.64, while the other had a value of 0.95; none had any documented clinical signs of residual neuromuscular block. Among the patients with upper respiratory tract infections, one had a final TOFR value of 1.44, and the other had a TOFR value of 0.91 with swallowing weakness in the recovery room. The sensibility for a < 0.91 to predict a lower respiratory tract complication (infection) was 50%, with a specificity of 69%, with a PPV of 5% and an NPV of 97% with an AR of 0.03, a RR of 2.17 and an OR of 2.23. The sensibility for a < 1.0 to predict a lower respiratory tract complication (infection) was 100%, with a specificity of 53%, with a PPV of 0.07 and an NPV of 100%, with an AR 0.07, and unable to determine RR and OR (because of a 0 false negative cases). All lower respiratory tract infections occurred in patients under 65 years with an AR of 0.04 and RR and OR of 0 each.

## Discussion

Current standards for quality care in anesthesia were first reported approximately in 2010 to increase patient safety during general anesthesia [7]. Many medical associations, including the Czech and French Societies of Anesthesiology, The Association of Great Britain and Ireland, stated the mandatory need for objective monitoring of neuromuscular block. They recommended a TOFR cut-off value of 0.90 for detecting residual NMB [7]. It is to highlight that the American Society of Anesthesiologists made no mandatory recommendation [7] on the use of NMB monitoring; in Mexico, there neither exists specific guidelines in this respect; nevertheless, it is common practice among our institution. We are proud to have a high acceptance rate among our anesthesia personnel for objective NMB monitoring, provided that all our institutional anesthesia equipment has integrated TOFR and that our institution participates in the High Proficiency Residence Program in anesthesia. Nevertheless, we are well aware that such an acceptance rate is not common practice worldwide yet [16]. Other problems, such as the availability of reversal agents, also play a role in mid to low-income countries such as ours as a cause of suboptimal treatment, an issue we will handle deep below.

The use of NMBA has significantly impacted current clinical practice for patients, anesthesiologists, and surgeons in many ways, including improved first-pass intubation for experts in elective conditions and non-experts in emergency conditions, aid for difficultness in facemask ventilation, enhanced surgical conditions for open and laparoscopic intermuscular approaches such as those of abdominal and pelvic procedures [17] in such a way that today NMB is considered vital for uneventful rapid sequence intubation [17].

Despite almost 80 years of experience in using NMB, several studies continue to report high incidence rates of residual neuromuscular block with the accompanying side effects and risks that it provides. Residual neuromuscular block increases the risk of pulmonary aspiration events and the hypoxic ventilatory response [14]. Respiratory complication rates reach 0.8% up to 6.5 of patients undergoing general anesthesia, which translates to 0.5 million up to 4 million worldwide yearly [3, 7]. Furthermore, the use of NMBDs has been repeatedly implicated in awareness during surgery when paralyzed patients have an inadequate level of anesthesia; such findings suggest that many clinicians may have an incomplete understanding of the pharmacology of NMBDs and of the existing techniques to monitor the level of the neuromuscular blockade after NMBD administration [14].

Mechanomyography (MMG) is the gold standard for monitoring neuromuscular function. Nevertheless, acceleromyography (AMG) is increasingly being used in a clinical setting as it is relatively inexpensive, easy to set up, and able to detect neuromuscular blocks with accuracy [15]. However, the baseline TOFR measured using AMG is significantly higher than MMG [15]. Authors used acceleromyograph (AMG) TOFR data, AMG- measured TOFR can exceed over 100. Raw AMG data have an idiosyncrasy. In contrast to MMG and electromyography (EMG) where the control (baseline) TOFR approximates 1.00 (100%), the control AMG-measured TOFR is more likely to be > 1.00 (> 100%). Values between 1.10 (110%) and 1.20 (120%) are common, and values of 1.40 (140%) are not rare [13, 15]. The current standard for “adequate recovery” from a neuromuscular block is the return of the TOFR to ≥0.9 measured at the adductor pollicis muscle, the cut-off point when an extubating is considered safe. Nevertheless, recent studies have suggested that several devices offer clinical raw TOFR data which can report values over 1 (or 100 in percentage) that require additional baseline calibration and suggest corrected values in the range of > 0.95–1, with trending to < 1.00 [4, 13, 15].

Additionally, to the possible incomplete understanding of the NMB effects and pharmacokinetics, a significant rate of underused monitoring is reported worldwide either by lack of devices, protocols, or culture to use monitoring [13, 16]. As considered above, this problem is complicated even more when there is difficulty acquiring trustable and sensible cut-off values [15]. Provided these issues, currently available devices are less than ideal, and expertise on their use is far from real [13].

Underdiagnosing RNMB is not surprising considering the lack of implementation and expertise on NMB monitoring and the variations in device parameters and cut-off criteria, which make unneeded reversal even plausible, as was the case in one of our cohort patients. Adding to the already high rate of RNMB poses a significant risk of complications, especially in high-risk populations such as older people and patients with serious comorbidities. Reversal agents significantly reduce this collateral damage; nevertheless, they are not without risk. Reversal agents can have serious detrimental effects in the postoperative period. Traditionally, we achieve the reversal of neuromuscular block by administering acetylcholinesterase inhibitors [14]. These drugs, such as neostigmine, have significant parasympathomimetic effects as acetylcholine interacts with cholinergic receptors [14]. For instance, these agents cause bradycardia and other bradyarrhythmias and bronchoconstriction through muscarinic receptor activation. In order to mitigate these effects, anti-muscarinic agents are co-administered with acetylcholinesterase inhibitors [14]. Once recovery is almost complete, administration of these agents may have the paradoxical effect of inducing muscle weakness [14]. Therefore untimely or wrong administration (as in the setting of an overestimated residual NMB) can produce instead of preventing serious pulmonary events.

Additionally, the type of reversal agent is also essential; there exists evidence that sugammadex reduces the risk of pulmonary complications by up to 30% [9]. Based on this information, we prefer using sugammadex at our institution, which is also in line with current US trends for NMB reversal during ENT surgery [2] . Sugammadex acts as a binding agent and has no effect on acetylcholinesterase [14]. Therefore, such reversal is devoid of the various side effects of acetylcholinesterase inhibition. Sugammadex, although safer, is not free from side effects, with hypersensitivity reactions occurring in 1 of 3500 cases. In such cases, cardiovascular collapse typically occurs within 4 minutes, urging for epinephrine and volume resuscitation [14].

Such problems make suboptimal treatment (underdiagnosed or overdiagnosed with unneeded reversal) account for preventable complications. Increasing the importance of these phenomena is the observed prolonged times of action of NMB agents in elderly populations [10]. Our study made a subanalysis demonstrating that patients under 65 behave as reported in theory with an effect of at least 60 minutes, while in our series, almost 74.51% of them exceeded 90 minutes. Nevertheless, none of the patients above 65 had below 90 minutes of deep NMB, with most of them having residual NMB (50.0 to 83.33% using TOFR criteria < 90 and < 1.00, respectively). Our series demonstrated these patients to have a 6.08-fold OR for increased residual NMB effect and are the population at higher risk of complications by this means derived from their age and comorbidities. The present study aids in demonstrating a significant increase in the rate of underdiagnosed residual NMB. For this reason, we should encourage the development of specific surveillance protocols and actions for this population to prevent significant complications, including the use of shorter-action NMB, early reversal, and prolonged surveillance in the postoperative period. Furthermore, we recommend improving quality control measures, including verifying device calibration and taking as much time as needed to have a trustable instrument.

Limitations, given the retrospective nature of the findings and the small sample size, we could not verify the process of TOF Baseline calibrations; nevertheless, it is a routine in our service practice.

## Conclusions

The rate of residual NMB in our practice was in the range of 29.9 to 49.1% depending on the criteria used (TOFR < 0.91 or TOFR < 1.00, respectively), more probably the higher limit, in line with literature ranges. We do recommend extubating patients based on a TOFR criterion of 1.00, especially on the elderly, to avoid clinically relevant residual NMB or its complications. Patients above 65 years old are at an increased risk of residual NMB (6.08 OR) due to increased length of action, as demonstrated by the curves of NMB during surgery. Older patients also had an increased risk (OR 11.75) of clinical symptoms related to the residual neuromuscular block. Therefore, we recommend providing specific surveillance protocols for patients above 65 years-old, including the use of shorter-action NMB, early reversal, and prolonged surveillance in the postoperative period. In addition, we recommend the use of reversal agents in patients at high risk of residual neuromuscular block, such as patients above 65 years-old, especially in those cases where patients have difficultness for reaching a TOFR criterion of 1.00 because of an increased risk of residual neuromuscular block and its complications. We will look to develop future research prospectively to corroborate these results.

### Supplementary Information

**Additional file 1.**

## Data Availability

All data is included as an additional file.

## Abbreviations

AMG: Acceleromyography
AR: Absolute Risk Ratio
ASA: American Society of Anesthesiologists score
CI: Confidence Interval
ENT: Ear-Nose-Throat
MMG: Mechanomyography
NMB: NeuroMuscular Block
NMBA: NeuroMuscular Blocking Agents
NPV: Negative Predictive Value
OR: Odds Ratio
PPV: Positive Predictive Value
PACU: PostAnesthetic Care Unit
RR: Relative Risk
SD: Standard Deviation
TOF: Train-Of-Four
TOFR: Train-Of-Four Ratio
US: United States
