# Correction: Rate of residual neuromuscular block using single‑dose rocuronium in general anesthesia for ENT Surgery: a retrospective cohort study

**DOI:** 10.1186/s12871-023-02309-4

**Published:** 2023-10-19

**Authors:** Orlando Carrillo-Torres, María Guadalupe Pliego-Sánchez, Víctor Joshua Pérez-Muñoz, Jennifer Sánchez-Jurado, Verónica Camacho-Vacherón, José Damián Carrillo-Ruiz

**Affiliations:** 1 Anesthesiology Service at Mexico General Hospital, Mexico City, Mexico; 2 Research Direction & Neurosurgery Service at Hospital General de México, Mexico City, Mexico; 3 Neuroscience Coordination of Psychology Faculty at Mexico Anahuac University, Av. Anahuac 46, Lomas Anahuac, Naucalpan de Juárez, Estado de México, 52786 Mexico

**Correction: BMC Anesthesiol 23, 107 (2023)**.

10.1186/s12871-023-02027-x.

Following publication of the original article [1], the authors reported that the last names of the authors are not correct as parts of the family names were captured as given names. The correct presentation of the authors names are as follows:

 Orlando Carrillo-Torres

María Guadalupe Pliego-Sánchez

Victor Joshua Pérez-Muñoz

Jennifer Sánchez-Jurado

Verónica Camacho-Vacherón

José Damián Carrillo-Ruíz

The author group has been updated above and the original article [1] has been corrected.
